# Time-lag of urinary and salivary cortisol response after a psychological stressor in bonobos (*Pan paniscus*)

**DOI:** 10.1038/s41598-021-87163-5

**Published:** 2021-04-12

**Authors:** Jonas Verspeek, Verena Behringer, Daan W. Laméris, Róisín Murtagh, Marina Salas, Nicky Staes, Tobias Deschner, Jeroen M. G. Stevens

**Affiliations:** 1 Behavioural Ecology and Ecophysiology Group, Department of Biology, University of Antwerp, Antwerp, Belgium; 2 Antwerp ZOO Centre for Research & Conservation (CRC), Royal Zoological Society of Antwerp (RZSA), Antwerp, Belgium; 3 Interim Group Primatology, Max Planck Institute for Evolutionary Anthropology, Leipzig, Germany; 4 Endocrinology Laboratory, German Primate Center, Leibniz Institute for Primate Research, Göttingen, Germany; 5 SALTO, Agro- and Biotechnology, Odisee University College, Brussels, Belgium

**Keywords:** Animal physiology, Neuroendocrinology, Mass spectrometry, Steroid hormones

## Abstract

Cortisol is often measured as a marker for stress. Therefore, a profound validation of the time-lag between the stressor and the increase and peak in cortisol levels is needed. No study measured both the urinary and salivary cortisol time-lag after a psychological stressor. In this study, we used a frequent sampling study design to (1) describe the urinary and salivary cortisol pattern during a control day; and (2) characterize the induced excretion pattern of urinary and salivary cortisol after a psychological stressor in six zoo-housed bonobos. Liquid chromatography-tandem mass spectrometry was used to analyze 71 urine and 162 saliva samples collected on a control and a test day. We found that the time-lag between the stressor and the maximal cortisol concentration was similar in urine and saliva (160 min after the stressor). However, salivary cortisol after the stressor did show a faster and steeper increase than urinary cortisol. We also show inter-individual variation in the baseline and stress levels of cortisol, which should be considered in future cortisol studies. Our research highlights the importance of validation studies to confirm relevant sampling windows for cortisol sampling in order to obtain biologically meaningful results.

## Introduction

Animals have developed behavioral and physiological adaptations to cope with stressors. One physiological response to a stressor is the activation of the hypothalamus–pituitary–adrenal (HPA) axis, which is a cascade of events, mediated by an integrated network of neuroanatomical structures and peripheral organs leading to physiological changes that help to restore homeostasis. This cascade is initiated after a stressor is perceived as such by the brain of an animal^1–3^. At first, activation of the paraventricular nucleus (PVN) of the hypothalamus results in the release of catecholamines (epinephrine and norepinephrine) from the peripheral nerves and the adrenal medulla of the sympathetic nervous system (SNS). Simultaneously, the neurons of the activated PVN in the hypothalamus secrete corticotropin-releasing hormone (CRH), which is transported through the hypophyseal portal system where it stimulates the anterior pituitary gland to release adrenocorticotropic hormone (ACTH) into the blood stream. In the course of minutes, ACTH stimulates the adrenal glands to release glucocorticoids (GCs). Following excretion, GC’s are transported in the circulation, either free or bound to proteins such as corticosteroid-binding globulin (CBG). The unbound GC’s are small and lipid-soluble, and can easily diffuse through lipid-rich cell membranes via passive intracellular diffusion. Once they arrive at the secretory endpiece of the salivary glands, these unbound GC’s pass through the cells into saliva^4,5^. The bound GC molecules are too large to leave the capillaries, and thus remain in circulation. The GC’s in circulation are transported to target tissues, where they bind to receptors in the brain, liver, kidneys and other tissues. This results in an array of effects, including the release of additional energy for necessary physiological functions to respond to the stressor. After exerting their function in these target tissues, GC’s are metabolized in the liver and kidney into compounds that are eliminated in urine and feces. Once the organism has responded to the stressor, high GC and ACTH levels activate a negative feedback loop, which inhibits the HPA axis and SNS cascade^1,2^. While this general stress-response is conservative across all vertebrate taxa, variations in the response exist due to many extrinsic (e.g. time of day, disease, social status) and intrinsic factors (e.g. species, body condition, reproductive status) (review in^6,7^). In measuring the individuals GC response, both the magnitude and duration are biologically relevant, since hormone-receptor interactions happen during the entire time of the response and not just at the maximum of the GC release^8^.

Cortisol is the main GC in many mammals and is therefore often used as a physiological marker of the stress response^9^. During days with minor HPA-axis challenges, secretion of cortisol from the HPA-axis follows a characteristic circadian pattern. In diurnal animals, cortisol and GC metabolite levels in plasma, urine and saliva are highest in the morning and decrease throughout the day^2,10–12^.

Stress-induced activity of the HPA axis can be measured in a variety of sample types and matrices, each having advantages and disadvantages (see reviews^6,7,13,14^). In brief, cortisol levels in blood represent a short-term stress response^4^, which allows for measuring the effect of a specific event on the activation of the HPA-axis. However, the handling and restraining of animals during blood collection can already be stressful and lead to an increase in cortisol levels^2,7,15,16^. Therefore repeated blood cortisol sampling within a short time frame is inadvisable. To avoid this confounding effect, the interest in non-invasive monitoring of cortisol levels in hair, feces, urine, and saliva has substantially increased^13^. Cortisol and GC metabolite levels in hair and feces reflect blood concentrations over months and days, respectively, and are useful to monitor cumulative and chronic aspects of stress responses^7,10,14,17^. However, to infer the effect of particular events on the activation of the HPA-axis, more short-term changes in cortisol levels need to be measured. Besides correlating averaged hormone concentrations with for example rates of a behavior across a time span^18–21^, one can also implement event-sampling. In event-sampling, hormone samples are collected within a certain time-frame following a single event and are expected to reflect the impact of this specific event^22–26^. Studies that use event sampling or short-term changes in cortisol levels, require a matrix reflecting those changes in time. Therefore, interest in urinary and salivary cortisol as non-invasive markers for stress has increased^27–30^. Urinary cortisol has been stated to reflect the longer lasting effect of a certain stressful event, since it represents the pooled cortisol values over minutes to hours, depending on the species^13,31,32^, while salivary cortisol concentrations reliably represent circulating cortisol levels with a short time-lag of several minutes^5,31,33,34^. To be able to measure the reactivity to a certain event, the sampling moment should be chosen based on the expected timing of the cortisol peak in both matrices. However, no study so far measured a provoked cortisol response after a psychological stressor using both urine and saliva sampling at comparable intervals. Frequent sampling of urine and saliva after such a stressor could provide a profound characterization of the time-lag of both cortisol responses, allowing for a comparison between them.

Cortisol levels can be measured using liquid chromatography-tandem mass spectrometry (LC–MS/MS), which is a technique that has been adapted recently to measure steroids in matrices other than blood. It combines the separation capabilities of high-performance liquid chromatography (HPLC) with those of a conjoined mass spectrometer^35^. Before using a method for biomarker analyses, a species-specific validation is needed since clear differences in metabolism and excretion of GC’s exist^10,13,36^. The use of LC–MS/MS has been validated to analyze steroid hormones in saliva in humans^37,38^ and chimpanzees^39^ and in urine in bonobos^40^, but not in saliva in bonobos.

Often physiological validation experiments, like an ACTH challenge, are used to induce artificial changes in circulating GC’s using a pharmacological stimulation of the HPA axis^10,14,41,42^. The injected ACTH directly stimulates the adrenals, circumventing the activation of all earlier steps of the HPA cascade^14,43^. After administration, GC levels increase and reach maximum values after a species- and matrix-specific time-lag. However, to ensure that the expected GC rise is strong enough to be detected by the method, often huge ACTH doses are used^6,14^. Such high ACTH stimulation will probably never occur naturally. This may be problematic, since peak characteristics like delay time, maximum and duration are shown to be dose dependent^44–46^. An alternative to detect the biologically relevant adrenal activity is to implement a biological validation. In this procedure, GC levels are monitored in a situation with an expected GC level change, for example the GC response before and after a stressful event. Such procedures offer a more valuable opportunity to validate assays since they provide biologically relevant information (e.g. response time, peak duration) about the actual stress response after a psychological stressor, which can be used in future studies. Because of their invasive character, physiological validation studies were often based on one or a few individuals^47–49^. However, inter-individual variation in the HPA-axis activity is well documented^6^. Ideally, validation studies should include more subjects to detect this variation hence avoiding generalizations based on only one individual response.

The objective of this study was to provide detailed information on the excretion pattern of cortisol in bonobos. Using frequent sampling of urine and saliva in multiple well-trained bonobos we aim to (1) describe the decreasing urinary and salivary cortisol pattern during a day without a stress response challenge to serve as a base-line (control); (2) characterize the induced urinary and salivary cortisol change after an acute, psychological stressor (test) to determine the time-lag of cortisol secretion increase and peak in urine and saliva. Finally, we aim to describe the difference between individual variation in the cortisol pattern during the control and test day in both matrices. Our study of the urinary and salivary stress response in bonobos can inform future research, in which event-sampling is used, to choose an adequate time window and matrix for sample collection.

## Methods

### Subjects and housing

We studied a group of bonobos at Zoo Planckendael (Belgium) (Table 1). During the study period, bonobos were housed in an enclosure consisting of nine interconnected rooms of various sizes (between 15 and 65 m^2^) which can be separated by sliding doors to allow temporary separation. Using positive reinforcement training, all bonobos were trained to regularly enter each of the rooms in family groups and individually. When being separated, individuals could always hear, smell and see other bonobos. Mothers were never separated from their dependent offspring. The shifting and individual separation is part of the normal morning routine of the bonobos, and therefore, are not considered as a stressor that could affect cortisol levels. The bonobos were fed four times a day and water was available ad libitum.

**Table 1:** Subject information.

Sex	Individual	Age (years)^a^	Amount of samples (successfully extracted/collected)			
			Control		Test	
			Urine	Saliva	Urine	Saliva
Female	Hortense	40	7/9	11/15	9/9	13/14
	Djanoa	23	7/9	12/15	7/7	7/8
	Nayoki	6	1/1	7/14	1/1	10/10
	Sakana^b^	1	–	–	–	–
Male	Vifijo	24	9/9	15/15	9/9	14/15
	Zamba	20	–	7/11	–l	13/15
	Habari	12	9/9	15/15	8/8	12/15
	Kikongo^b^	4	–	–	–	–

^a^Age: individual’s age when the study took place.

^b^Did not provide any samples in this study.

### Sample collection

Before the onset of this study, five subjects had been trained to deliver urine samples into cups and trays. Six bonobos had also been trained to participate in saliva sample collection using Salivettes (Ref 51.1534, Sarstedt, Nümbrecht, Germany) (Table 1). Samples were collected during two days. On the control day, all independent subjects were housed individually from 9:50 until 12:00 pm. For management reasons, at 12:00 pm the family groups were reunited. Urine samples were taken once every hour between 9:50 and 18:00 h. Saliva samples were taken at short intervals (every 15 to 20 min) between 9:50 and 12:00 pm. From 12:00 to 18:00 h, we prolonged the sampling interval for saliva samples to once every hour, simultaneously with the urine sample collection. Exactly 1 week after the control day, we collected samples on the test day to determine the effect of the stressor on urinary and salivary cortisol levels. The procedures of housing and sampling were identical to those described for the control day. The only difference on this second day was the arrival of the zoo veterinarian in the building at 10:18 h, which is perceived by all individuals as a stressor. In the past, all bonobos had experienced being sedated with a blowpipe by the veterinarian for medical controls or for transfer between enclosures. Previous experience had shown they responded to the presence of the veterinarian by showing behavioral indicators of being stressed, such as increased locomotion, uttering alarm calls or aggressive vocalizations, increased anxiety behaviors (auto-scratching), and the secretion of diarrhea. Therefore, the presence of the veterinarian with a blowpipe is perceived as a threat by all bonobos, and is assumed to be an appropriate stressor in their zoo environment^50^. After the veterinarian had entered the building, he carried his blowpipe in his hand, stopped in front of each enclosure, and called each bonobo by its individual name. All bonobos responded in the usual way by moving around, seeking comfort from group members (if housed in subgroups) and uttering alarm calls. After 2 min the veterinarian had visited every bonobo in the mentioned procedure and left the building. The control day was implemented to control for possible diurnal effects and to show that the result in the test day was caused by the stressor rather than the sampling regime.

Urine samples (N = 71, mean ± SD = 7.1 ± 3.1 samples/individual/treatment, range 1–9 samples/individual) were directly taken from the cups and trays using a syringe. Next, urine samples were stored in Cryo-vials (Ref. E292.1, Roth, Karlsruhe, Germany) at − 20 °C until analysis. Saliva samples (N = 162, mean ± SD = 13.5 ± 2.3 samples/individual/treatment, range 8–15 samples/individual) were taken by sweeping the Salivette through the mouth of each subject which took approximately 30 s. After collection, saliva samples were immediately placed in Salivette tubes (Ref 51.1534, Sarstedt), and also stored at − 20 °C until analysis. To minimize differences in sampling time between individuals, saliva was collected by four and urine by five keepers and researchers, who were all familiar to the bonobos. Throughout the control day and the test day, every bonobo was always sampled by the same experimenter to avoid possible bias.

#### Sample preparation

##### Urine sample preparation and measurement

The extraction was done following the extraction protocol of Hauser et al. with adaptions explained in Wessling et al. An internal standard mix, containing 250 ng/ml d3-testosterone, d4-estrone, d9-progesterone and d4-cortisol, was added to each sample as a quality control. Samples were re-measured if the internal standard recovery deviated by more than 80% of the internal standard. Urinary hormone levels need to be adjusted for variable water content among spot urine samples, which depends on the hydration status of an individual and the time since last urination. Therefore, to compensate for variation in urine concentration, we measured the specific gravity (SG) using a digital handheld refractometer (TEC, Ober-Ramstadt, Germany) and calculated urinary cortisol corrected for SG^51^. The SG population average was 1.006. We successfully extracted 67 urine samples. Urinary cortisol levels were measured using LC–MS/MS following Wessling et al. (2018) and quantified with MassLynx (version 4.1; QuanLynx-Software).

##### Saliva sample preparation and measurement

The frozen Salivettes were thawed in the salivette tubes (ref 51.1534, Sarstedt). 100 µl of clear supernatant were transferred into a SafeSeal tube (Multi SafeSeal tubes, 7080.1, Roth) before adding 5 µl of internal standard working solution consisting of internal standard mix (containing 250 ng/ml d3-testosterone, d4-estrone, d9-progesterone and d4-cortisol, e. g. 98%, Prod. No. D-5280, CDN Isotopes) and 100 µl of methanol/ZnSO_4_ (50 mg/ml). The sample was vortexed then centrifuged at 12,000 rpm for 5mins. Prior to solid phase extraction (SPE) the supernatant was diluted with 1 ml water. HR-X cartridges (HR-X, 1 ml/30 mg, ref 730934 Chromabond) were conditioned with 1 ml methanol followed by 1 ml water. After transferring the supernatant to a cartridge, it was washed with 1 ml water. Steroids were eluted with 1 ml methanol followed by 1 ml ethylacetate. They were dried down under a stream of compressed air at 45 °C and reconstituted in 100 µl of 30% acetonitrile for LC–MS/MS analysis. To measure salivary cortisol, the same LC–MS/MS procedure was used as described for urinary cortisol^52^. We successfully extracted 136 saliva samples. Samples were re-measured if the internal standard recovery deviated by more than 80% of the internal standard.

### Method evaluation

For the chemical validation of the LC–MS/MS assay, we used human saliva samples since the available amount of bonobo samples was not enough to perform the necessary validations. To assess the accuracy of the cortisol measurement by the LC–MS/MS assay, two pools of human saliva samples were created: one for men (N = 5) and one for women (N = 5). Each pool was spiked with two different concentrations of internal standard solution (low and high). A 200 µl aliquot of each of the pools, spiked pools, and internal standard solutions were extracted five times and measured by LC–MS to determine extraction efficiency. Average recovery of the saliva samples spiked with the high concentration of the internal standard solution was 100.50% (SD = 6.68) for the males and 98.20% (SD = 4.64) for the females. With the low concentration, average recovery was 101.02% (SD = 3.98) for the males and 105.34% (SD = 5.35) for the females (see Supplementary Table S1). The difference between repeated extracts of each sample group was lower than 7% (see Supplementary Table S2). Internal standard deviation was acceptable (< − 60%) for all extracts (see Supplementary Table S3).

### Statistical analyses

To explore differences in cortisol concentrations between the control and the test day after the arrival of the zoo vet, we used linear mixed models (LMM) using the “lmer” function from the “lme4” package^53^. On the test day, the amount of samples that were collected prior to the arrival of the stressor (one urine and two saliva samples) didn’t allow for statistical testing. Therefore, only cortisol concentrations after the arrival of the stressor were compared. To examine the influence of the within-subject predictor variables (a) sampling time, (b) the quadratic term of sampling time and (c) treatment (control or test) on urinary and salivary cortisol concentrations, we ran two LMMs: one for the urine samples (urinary stress response model) and one for the saliva samples (salivary stress response model). In addition to the main effects, we included all two-way interactions between the main effects as fixed effects. We also included subject-ID as random intercept and sampling time and treatment as random slopes. For all models, we used diagnostic plots (residuals vs fitted and qqplot) to examine assumptions of normality and homogeneity of variances, and we tested uniformity and dispersion of the residuals using the “DHARMa” package^54^. To meet assumptions of homoscedasticity and normality of residuals, we log-transformed the cortisol data. Model stability was assessed by excluding random effects and comparing the estimates derived with those derived for the full data set, indicating no influential random effects. Significance of the fixed effect was determined by comparing the full model with the respective null model, excluding the fixed effect but retaining the random effects, using a likelihood ratio test (“anova” function in R^55^). All statistical analyses were done using R 3.3.2^56^ and plots were generated using the statistical package “ggplot”^57^.

### Ethical statement

Animals were never harmed in any way throughout the duration of this study and participation in sample collection was voluntary. Urine and saliva were collected using standard non-invasive methods. The care and housing of all bonobos was adherent to the guidelines of the EAZA Ex-situ Program (EEP). All research complied with the ASAB guidelines^58^ and was carried out in accordance with the national regulations. This study, including all experimental protocols, was approved by the Scientific Advisory Board of the Royal Zoological Society of Antwerp and Zoo Planckendael (EC-3/SGZ(10-12-19)) and The University of Antwerp (Belgium). Informed consent was obtained from all human participants included in this study.

## Results

### Urinary stress response model

**Figure 1:**
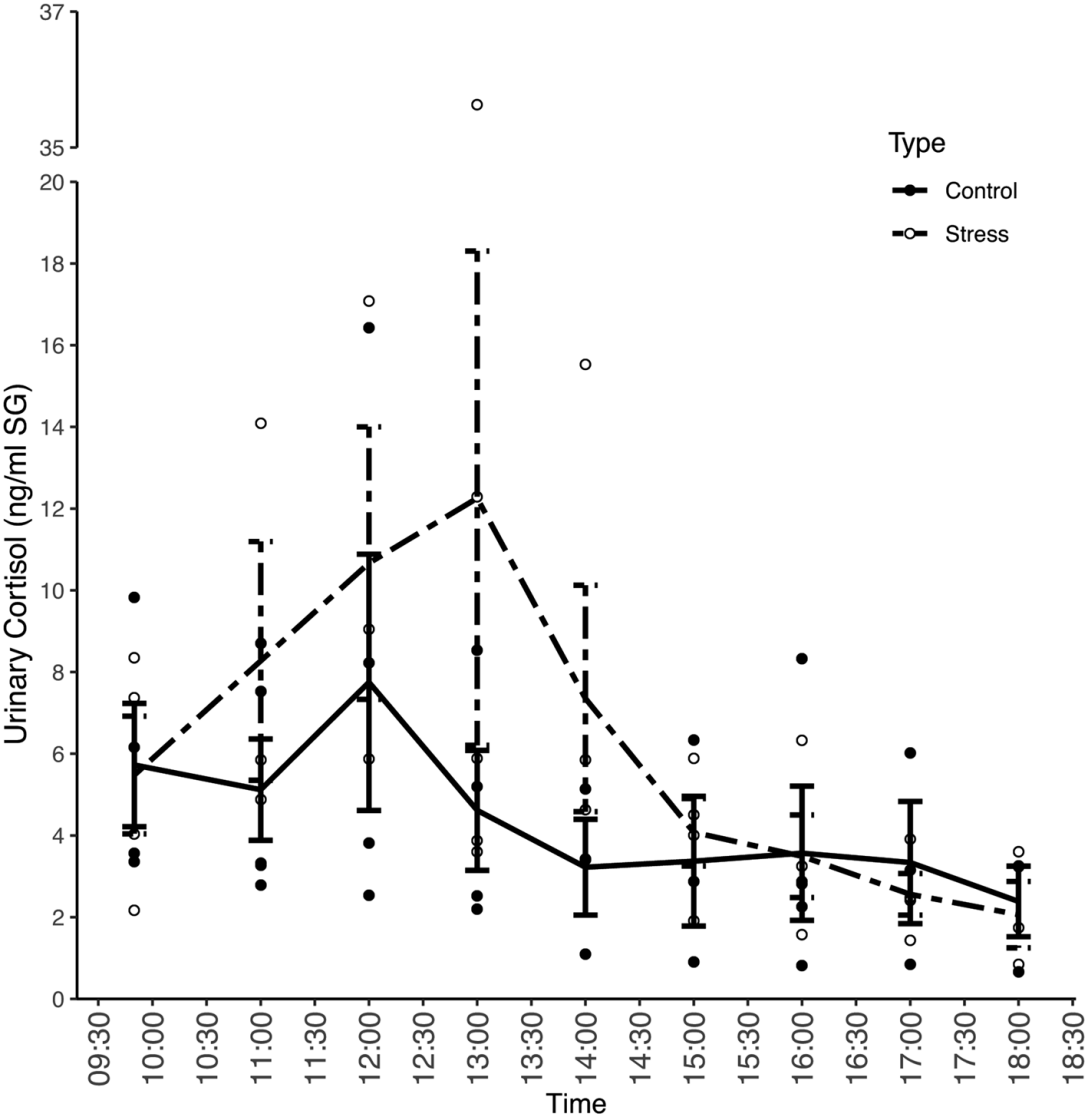
Mean (+ SEM) urinary (N = 108) cortisol levels in bonobos during the control day (solid line) and stress day (dashed line). The local veterinarian (the stressor) entered the building at 10:18 h. The analyses were conducted on log transformed cortisol data but actual values are displayed here to provide better visual and interpretable representation of the cortisol response.

We compared urinary cortisol levels on the control day (mean 4.52 ng/ml corr. SG; range 0.6–16.42 ng/ml corr. SG) and test day (mean 6.36 ng/ml corr. SG; range 0.84–35.63 ng/ml corr. SG). During the control day, the average urinary cortisol levels showed the expected diurnal decline. However, a peak occurred around 12:00 h (Fig. 1, solid line). On the test day, when subjects were exposed to the acute stressor, the characteristic diurnal pattern was disrupted, and the urinary cortisol pattern differed from the control day pattern. In contrast to the declining cortisol levels on the control day, after the stressor, mean urinary cortisol levels steadily increased within 40 min and reached a maximum level that doubled the control levels after 160 min (13:00 h: 12.26 ± 5.41 pg/µl). Afterwards, urinary cortisol levels declined and reached control levels 340 min (16:00 h) after the arrival of the stressor (Fig. 1, dashed line). Statistical analysis showed that urinary cortisol levels throughout the day showed a different pattern between the control and test day (interaction term of sampling time and treatment: χ^2^ = 6.00, df = 1, P = 0.01) (Table 2).

**Table 2:** Results of the Linear Mixed Model comparing the urinary cortisol levels between the control and test day.

	Estimate	Std. error	t value	P
Urinary stress response model				
Intercept	2.01	0.23	8.91	
Treatment^a^	1.02	0.38	2.70	0.13
Sampling time	− 0.17	0.05	− 3.58	** < 0.001**
Sampling time^2^	− 0.02	0.01	− 1.36	0.18
Sampling time × treatment	− 0.11	0.05	− 2.45	**0.014**
Sampling time^2^ × treatment	0.004	0.02	0.16	0.88

^a^Reference category for treatment was set to control.

Boldface indicates significance with P < 0.05.

### Salivary stress response model

**Figure 2:**
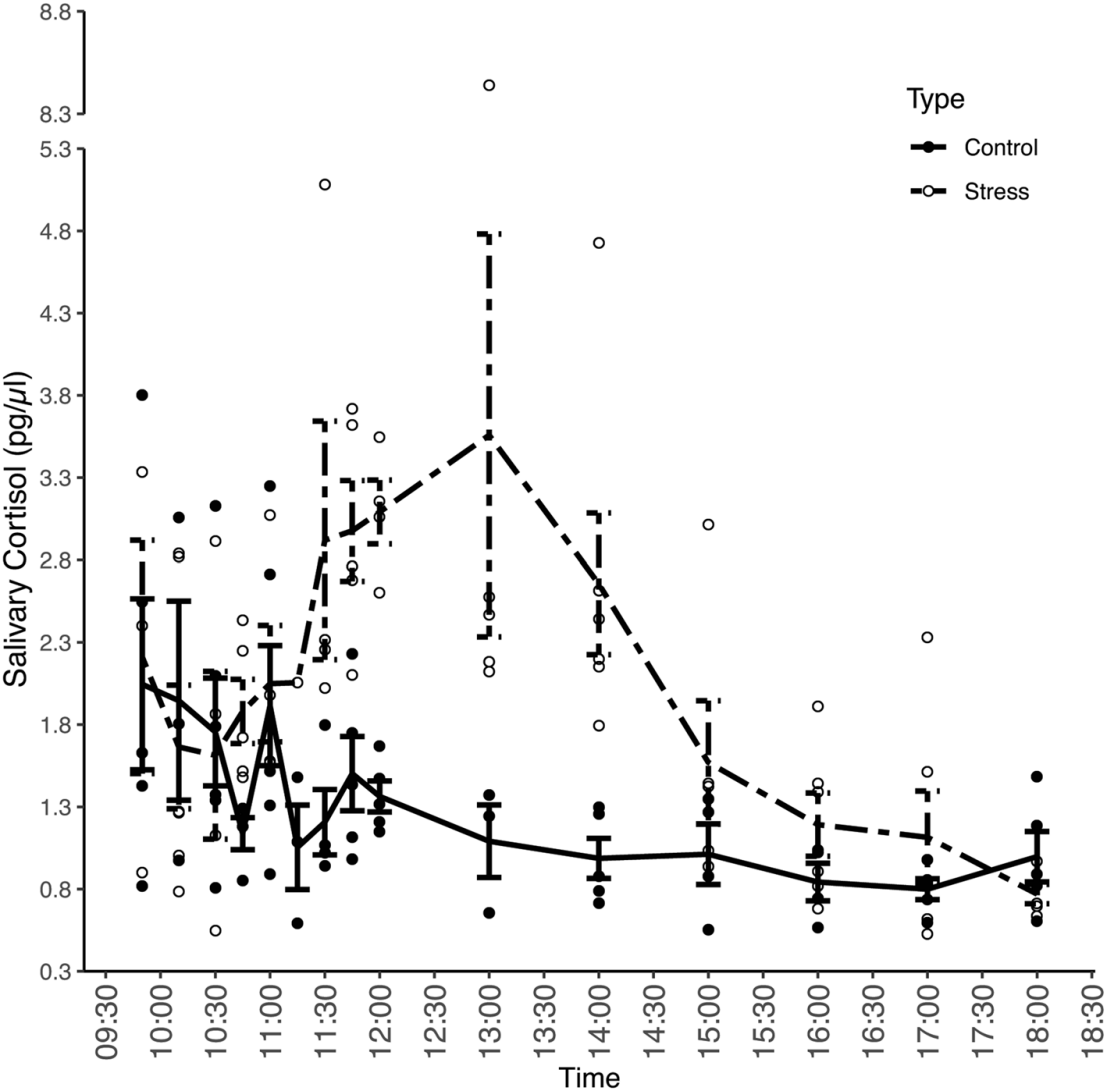
Mean (+ SEM) salivary (N = 136) cortisol levels in bonobos during the control day (solid line) and stress day (dashed line). The local veterinarian (the stressor) entered the building at 10:18 h. The analyses were conducted on log transformed cortisol data but actual values are displayed here to provide better visual and interpretable representation of the cortisol response.

We also compared salivary cortisol levels between the control (mean 1.34 pg/µl; range 0.55–3.80 pg/µl) and test day (mean 2.04 pg/µl (range 0.53–8.44 pg/µl). As in the urine samples, salivary cortisol showed the expected declining pattern during the control day. After the exposure to the acute stressor, salivary cortisol levels showed a fast increase within 25 min after the stressor, and reached maximum levels after 160 min (13:00 h: 3.56 ± 1.09 pg/µl) which is comparable to urinary cortisol levels. Within 30 min, salivary cortisol levels reached levels more than twice as high as the control levels and remained elevated above control levels for more than 240 min (Fig. 2). Statistical analysis showed that the salivary cortisol pattern throughout the day differed between the control and test day (sampling time × treatment: χ^2^ = 38.699, df = 1, P < 0.001) and that the increase and decrease of salivary cortisol levels throughout both days differed (interaction term of sampling time^2^ × treatment: χ^2^ = 41.423, df = 1, P < 0.001) (Table 3).

**Table 3:** Results of the linear mixed model comparing the salivary cortisol levels between the control and test day.

	Estimate	Std. error	t value	P
**Salivary stress response model**				
Intercept	0.59	0.26	2.23	
Treatment^a^	− 1.42	0.35	− 4.08	** < 0.001**
Sampling time	− 0.04	0.06	− 0.68	** < 0.001**
Sampling time^2^	− 0.0008	0.003	− 0.26	** < 0.001**
Sampling time × treatment	0.52	0.08	6.22	** < 0.001**
Sampling time^2^ × treatment	− 0.03	0.005	− 6.44	** < 0.001**

^a^Reference category for treatment was set to control.

Boldface indicates significance with P < 0.05.

### Individual urinary and salivary cortisol patterns

**Figure 3:**
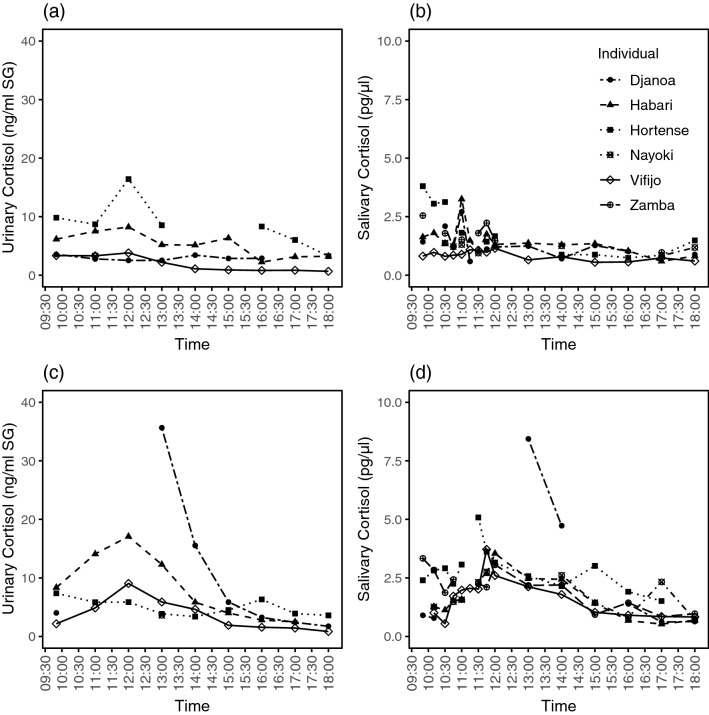
Individual cortisol levels in (**a**) urine during the control day; (**b**) saliva during the control day; (**c**) urine during the test day; and (**d**) saliva during the test day. In the test day, the stressor entered the building at 10:18. The analyses were conducted on log transformed cortisol data but actual values are displayed here to provide better visual and interpretable representation of the cortisol response.

We also present the inter-individual variation in urinary and salivary cortisol levels (Table 4, Fig. 3a,b). Individuals with the lowest urinary cortisol control levels also had the lowest salivary cortisol control levels. With the exception of one individual (Hortense), a similar pattern can be seen in the cortisol levels during the test day: individuals with a strong cortisol response in urine, also showed a strong cortisol response in saliva; and the individual with the lowest urinary cortisol peak also showed the lowest salivary cortisol peak (Fig. 3c,d). In the subject with the aberrant cortisol pattern (Hortense), higher urinary cortisol levels were found during the control than the test day and no urinary stress response was found during the test day.

**Table 4:** Mean (± SEM) urinary and salivary cortisol concentrations per individual.

	Urinary cortisol (ng/ml corr. SG)		Salivary cortisol (pg/µl)	
Individual	Control	Test	Control	Test
Hortense	8.73 ± 1.41	4.97 ± 0.45	1.79 ± 0.31	2.81 ± 0.24
Vifijo	1.89 ± 0.41	3.60 ± 0.85	0.84 ± 0.05	1.66 ± 0.22
Djanoa	2.94 ± 0.14	9.78 ± 4.31	1.29 ± 0.16	2.50 ± 1.05
Zamba	–	–	1.76 ± 0.18	2.04 ± 0.21
Habari	5.25 ± 0.64	8.37 ± 1.83	1.42 ± 0.15	1.66 ± 0.26
Nayoki	3.27	3.6	1.16 ± 0.07	1.73 ± 0.22

## Discussion

This study provides detailed information on the excretion pattern of urinary and salivary cortisol in bonobos. First, we found the expected decreasing pattern of urinary and salivary cortisol throughout the control day and, secondly, we characterized the increase in urinary and salivary cortisol response after an acute stressor.

As an anticipatory response to awakening, the highest cortisol production occurs in the second half of the night with peak cortisol values in the early morning^12,59–62^. Thereafter, cortisol values decline throughout the day^2,10,11^. Our results also showed the characteristic decreasing pattern of urinary and salivary cortisol, which is in line with previous studies in bonobos that used LC–MS/MS to measure urinary cortisol^63^ and enzyme immunoassays (EIA) to measure salivary cortisol^64–66^. In the present study, sampling was not possible before 9:20 h, so we were not able to include the early morning cortisol levels, which might have shown an even stronger effect of the awakening response. The cortisol levels measured in our study are comparable to urinary and salivary cortisol levels in humans^37,67^ and chimpanzees^39,68^ measured with LC–MS/MS but lower than salivary cortisol levels in bonobos measured with EIA^65^ and RIA^69^. This is not surprising given that these assays not only measure the native hormone but also their metabolites due to cross reactivities of their antibodies^13,70^. As extraction efficiency in our study was within the previously reported acceptable range of 80–120%^71^, our results demonstrate that LC–MS/MS can reliably detect known physiological patterns in bonobo saliva.

While urinary and salivary cortisol levels decreased throughout the day, we also found some slight deviations from this overall pattern: a small cortisol increase was found in both urine (12:00 pm) and saliva (11:45 h). These deviations might be due to excitement in anticipation of husbandry routines like feeding. Previous studies showed that in addition to the photoperiod, several other stimuli like feeding or social cues can alter the decreasing cortisol pattern in mammals^11,43,64,72^. Animals have evolved predictive homeostatic mechanisms and utilize specialized functions of the circadian timing system that enable them to, for example, predict the availability of food whenever it is consistently available at a specific time each day^5,73–76^. The short cortisol peak around 12:00 pm in both urine and saliva might therefore be the anticipatory response of the HPA-axis to the daily feeding moment between 11:00 and 11:30. More research is needed to verify whether this anticipatory effect is present so that future studies can take this into account when planning sampling intervals.

We also measured urinary and salivary cortisol levels after an acute stressor, the zoo veterinarian in this case. Since previous encounters between the veterinarian and these bonobos resulted in increased behavioral indicators of stress (e.g. locomotion, alarm calls, scratching), we expected to find a clear cortisol response after the stressor in both urine and saliva. Exposure to the stressor resulted in a significant increase in urinary and salivary cortisol. Moreover, we found that the urinary cortisol pattern throughout the day differed between the control and the test day. While control cortisol levels showed an overall decrease (see above), a cortisol peak was found during the test day. Surprisingly, the maximal urinary cortisol levels were reached within 160 min after the stressor, which is considerably shorter than 5.5 h (330 min) reported for peak excretion of cortisol in primates^47^. This difference might be explained by the different settings. The Bahr et al.^47^ study administrated radio-labeled cortisol, while we used a psychological stimulus to initiate a stress response. Moreover, the Bahr et al.^47^ study collected urine samples opportunistically of one individual of three primate species. In our study, samples were collected frequently at regular intervals in multiple well-trained subjects of the same species. And finally, primates in the Bahr et al.^47^ study were housed in metabolic cages during the collection time, whereas bonobos in our study were able to show locomotor activities. Differences in the cortisol response between our data and the previous study^47^ are expected since cortisol accumulates in urine over time and the cortisol levels in excreted urine are therefore dependent on the frequency of urination. In this previous study, the first chimpanzee sample was collected 2 h after injection^47^ so no conclusions could be made about the earlier cortisol excretion pattern. Moreover, the first urine sample already contained the second largest radio-labeled cortisol concentration, which might be the result of the accumulation of urinary cortisol over time. In case opportunistic and irregular sampling is used, cortisol levels in urine accumulate over an unknown time frame. We avoided this by implementing regular and shorter sampling intervals so that cortisol levels in urine always accumulate over the same time. This results in more standardized urinary cortisol levels per interval and a urinary cortisol response that represents the actual plasma levels over time. Using more frequent sampling, the time window of the maximal urinary cortisol value after a stressor in great apes was shortened from 24 h^77^ to 4.5 h^47^ and to 160 min in our study.

For salivary cortisol, we also found that exposure to a stressor significantly increased cortisol levels. Salivary cortisol rose rapidly (between 10 and 40 min after the stressor) and reached levels to more than double the control levels. This is in line with a previous study on common marmosets, where translocation to a novel environment resulted in cortisol levels that doubled the levels obtained prior to the translocation^78^. Previously in bonobos, salivary cortisol has also been found to reach levels nearly twice as high as normal after a parturition event^65^, after the transfer to a new building and after the integration of a new female in the group^79^. Surprisingly, the post-stress cortisol peak in our study only reached its maximal level after 160 min, and only returned to baseline levels 5 h later. In humans, salivary cortisol rose 1–3 min after a cortisol injection^4,80^ and reached its maximum levels after 35 to 40 minutes^5,81^. Also in one chimpanzee, the maximal salivary cortisol level after an ACTH challenge was reached after 45 min^28^. We had expected a delay of salivary cortisol rise when comparing with such physiological validation studies since the injection of ACTH immediately stimulates the adrenals, circumventing earlier steps in the HPA cascade^14^. A psychological stressor activates the HPA axis from the start, likely resulting in a longer time-lag of the cortisol peak. A pertinent question in the psychoneuroendocrinology of cortisol is to what extent the time courses of the cortisol responses to pharmacological stimuli differ from physical and psychological stressors^4,82–84^, and how different types of stimuli may recruit various aspects of the HPA cascade to different degrees^3^. The longer time-lag after a psychological stressor in our study in comparison with the time-lag after the pharmacological stimulation in the previous study on one chimpanzee^28^ indicates that the time frame of the cortisol response differs between physiological and psychological stimuli. Not only the timing, but also the amplitude of the salivary stress response differs significantly between our study and the previous chimpanzee study^28^. In the chimpanzee, salivary cortisol levels after the ACTH stimulation reached peak levels that were at least three, but for most individuals more than five times higher than the maximal levels we measured in bonobos. In response to a stressor, the extent of ACTH release is limited by a rapid feedback mechanism^3^. In physiological validation studies however, the sudden rise of plasma ACTH levels might not only stimulate faster cortisol secretion, but also immediately activate a strong negative feedback loop, possibly resulting in a high but shortened cortisol peak. In our study, the ACTH levels might have possibly increased more gradually after the activation of the HPA-axis in response to the psychological stressor, and this might have resulted in a delayed feedback and therefore a longer cortisol response compared to physiological validation studies. However, to really compare the two settings and to exclude species-specific differences, future study designs should compare both methods in different species using regular sampling on multiple subjects.

In comparison with humans, where salivary cortisol levels after a social stress test reached baseline levels after 70 min^85^, it is surprising that salivary cortisol levels in our study remained elevated for 5 h after the maximal value. However, a previous study reported that salivary cortisol levels after the birth of a bonobo infant remained elevated for more than seven and a half hours^65^. The arrival of the veterinarian in this study might have had a strong effect on the HPA axis activation resulting in a broader salivary cortisol response. Given that the magnitude and duration of the cortisol response reflect the strength of the stressor^7^, we suggest that the veterinarian indeed is a potent stressor that causes a measurable salivary cortisol peak in bonobos.

Surprisingly, the timing of the urinary and salivary cortisol peak after the stressor was found to be very similar. The maximal average cortisol level in both matrices was reached after 160 min and a similar decline in cortisol levels was found. This is surprising since a longer time-lag in urinary cortisol is expected^31^. However, our data show a faster and steeper increase in salivary than urinary cortisol levels. This is in accordance with previous literature stating that cortisol in saliva rises faster than in urine^31^. While the longer rise in salivary cortisol might be the result of the strong effect of the stressor, the urinary stress response does not show such a long peak. Further research comparing the cortisol response to a psychological stressor in both matrices is needed in order to help explain this unexpected result. In addition to the strong effect of the stressor, the anticipatory effect to the feeding moment around 12:00 might have influenced the urinary and salivary cortisol response. To avoid such confounding effects, future studies should not only take the diurnal cortisol pattern but also the effect of husbandry routines into account. We suggest future research to monitor the responses to routine handlings by including control measurements, as we have done in this study. Ideally, experiments involving cortisol measures should be conducted outside of these responses and in the afternoon when cortisol levels and intra-individual variability are lower^61^.

We also compared the individual cortisol levels during the control and test day. The inter-individual differences we found might partly be due to the large age variation of the subjects. While cortisol concentrations did not differ between male and female bonobos^86,87^, cortisol levels gradually increased during the ontogeny of wild bonobos^87^. The higher control values of the oldest female could therefore be the result of an age effect. We also found that individual differences in the cortisol levels were consistent across both matrices. Bonobos with low urinary cortisol levels during the control day, had low salivary cortisol levels during the control day and individuals with a lower urinary cortisol peak in the test day also showed a lower salivary cortisol peak during the test day. However, no clear pattern was found across treatment days. While some individuals with low cortisol levels on the control day also showed a lower cortisol peak after the stressor, another individual with low cortisol levels during the control day showed the highest cortisol stress response in both urine and saliva. The inter-individual variability was larger in the urinary than the salivary cortisol levels, which could be due to metabolic differences between individuals since urinary cortisol is metabolized before excretion^1,4^. While overall we found a decreasing pattern of urinary and salivary cortisol levels throughout the control day, we showed individual differences in the amount of cortisol but also in the fluctuations of the cortisol patterns. For the stress response, we found that the time frame of the peak was very similar but that the magnitude showed inter-individual differences. These differences are not necessarily surprising given that individual variation in the HPA axis activity is well documented (review in^6,7^). Moreover, these individual differences are consistent across both matrices: individuals with a strong urinary cortisol response also show a strong salivary cortisol response. Our findings complement previous results on the existence of individual stress reactivity, which has been linked to differences in behavior, neurobiology and immune response^88,89^. A study on the link between the individual stress responses and personality in a large group of bonobos could be a fascinating topic for future research.

The fact that in one of the subjects a salivary but not an urinary stress response was found, is surprising given that both matrices reliably reflect plasma cortisol levels^13^. Also, in this subject, the highest average cortisol levels during the control day were measured. The absence of a urinary cortisol peak in combination with the higher control levels might be indicative of underlying issues with the homeostatic regulation of this individual. These individual variations in the cortisol pattern during the control and the stress day show the importance of including more than one subject in validation studies and of using individuals as their own control using a repeated measures design^44,90^.

Saliva sampling has been shown useful to monitor animal welfare (e.g.^91,92^) but also in relation to cognitive tasks (e.g.^93^). Our data support previous research that showed that in bonobos salivary cortisol can be used to monitor short-term effects of stressful events^69^. In addition, we show that urinary cortisol can also be used to monitor the effect of an acute stressor. However, in order to find a physiological effect of a certain event, the appropriate time window after the event for sample collection should be chosen based on species-specific excretion patterns in a biologically relevant setting. We therefore suggest that instead of only taking physiological validation results into account, future endocrinological research should also consider biological validation studies when deciding on sampling intervals. Alternatively, studies could conduct a pilot study in which they collect consecutive samples to determine the ideal time window for sample collection after a specific event. Another approach, is to take the urination interval into account in which cortisol levels are compared between samples before and after an event^23^. However, in this case, cortisol values are accumulated over a longer period so solutions need to be developed to take this problem into account.

In conclusion, this study provides detailed information on the urinary and salivary cortisol response after a psychological stressor in bonobos. We show that saliva and urine can be used to monitor the cortisol response after an event but also that the time frame of sampling is crucial in order to obtain biologically relevant information. When designing research plans, we suggest future endocrinological studies to consider information from a relevant biological context to decide on the ideal time frame of sampling.

## Supplementary Information

Supplementary Information.

## Data Availability

The datasets analyzed during the current study are available from the corresponding author on reasonable request.
