# Supplementary material for: Time-lag of urinary and salivary cortisol response after a psychological stressor in bonobos (Pan paniscus)
Source: Sci Rep. 2021 Apr 12;11:7905. doi: 10.1038/s41598-021-87163-5 (PMC8041877; doi:10.1038/s41598-021-87163-5)
Supplement: Supplementary file 1 — Supplementary Information. [file 41598_2021_87163_MOESM1_ESM.pdf]

Electronic supplementary material

Time-lag of urinary and salivary cortisol response after a psychological stressor in bonobos (*Pan paniscus*)

Jonas Verspeek[1,2*], Verena Behringer[3,4], Daan W. Laméris[1,2], Róisín Murtagh[3], Marina Salas[2], Nicky Staes[1,2],Tobias Deschner[3], Jeroen M. G. Stevens[1,6]

1 Behavioural Ecology and Ecophysiology Group, Department of Biology, University of Antwerp, Antwerp, Belgium.

2 Antwerp ZOO Centre for Research & Conservation (CRC), Royal Zoological Society of Antwerp (RZSA), Antwerp, Belgium.

3 Max Planck Institute for Evolutionary Anthropology, Interim Group Primatology, Leipzig, Germany

4 Endocrinology Laboratory, German Primate Center, Leibniz Institute for Primate Research, Göttingen, Germany

5 SALTO, Agro- and Biotechnology, Odisee University College, Belgium

*Corresponding author's e-mail address: jonas.verspeek@uantwerpen.be

Supplementary Table S1: Extraction efficiencies of the spiked pool samples of men and women (n = 5)

Sex	Spike conc.	Extraction efficiency (%)	Standard deviation
**Men**	Low	101.02	3.98
	High	100.45	6.68
**Women**	Low	105.34	5.35
	High	98.20	4.64

Supplementary Table S2: Coefficient of variation of the extracts (n=5)

	Spike conc.	Cortisol
**Internal std**	Low	1
**Internal std**	High	6
**Male pool**	*Not spiked*	6
**Female pool**	*Not spiked*	2
**Male pool**	Low	1
**Male pool**	High	3
**Female pool**	Low	4
**Female pool**	High	1

Supplementary Table S3: Internal standard deviation of the extracts (n = 5)

	Spike	Cortisol-d4
**Male**	*None*	-43.7
**Male**	*None*	-52.4
**Male**	*None*	-52.7
**Male**	*None*	-54.1
**Male**	*None*	-47.1
**Female**	*None*	-49
**Female**	*None*	-52.3
**Female**	*None*	-45.3
**Female**	*None*	-52.8
**Female**	*None*	-50.1
**Male**	Low	-52.3
**Male**	Low	-47.9
**Male**	Low	-47.5
**Male**	Low	-51.3
**Male**	Low	-48.5
**Male**	High	-51.6
**Male**	High	-51.4
**Male**	High	-44.3
**Male**	High	-48.1
**Male**	High	-44.6
**Female**	Low	-49.6
**Female**	Low	-51.2
**Female**	Low	-52.3
**Female**	Low	-51.2
**Female**	Low	-42.2
**Female**	High	-44.5
**Female**	High	-44.3
**Female**	High	-51.1
**Female**	High	-53.3
**Female**	High	-43.6
